# Vaginal delivery after caesarean section and its associated factors in Mizan Tepi University Teaching Hospital, Southwest Ethiopia

**DOI:** 10.1016/j.heliyon.2021.e08276

**Published:** 2021-10-27

**Authors:** Yeabsira Girma, Zerihun Menlkalew, Alemnew Destaw

**Affiliations:** a Center for a Family Service Organization, Addis Ababa, Ethiopia; b LeDeG Midwifery College, Addis Ababa, Ethiopia; c Department of Epidemiology and Biostatistics, School of Public Health, College of Medicine and Health Science, Mizan- Tepi University, Mizan-Teferi, Ethiopia

**Keywords:** Vaginal delivery, Caesarean section, Success rate, Ethiopia

## Abstract

**Background:**

Vaginal birth after caesarean section is appropriate for a majority of women who have had a single prior lower segment caesarean section. However, little is known about vaginal birth after caesarean section in Ethiopia. Thus, this study aimed to assess the success rate of vaginal birth after caesarean section and its associated factors in Mizan-Tepi University Teaching Hospital, southwest Ethiopia in 2020.

**Methods:**

An institutional-based cross-sectional study was conducted among 416 mothers who gave birth by caesarean section from 2017 to 2019. The data were entered into epidata version 3.1.0 and analysed by SPSS version 21.0. Multivariable logistic regression analysis was performed to identify the factors associated with successful vaginal birth after caesarean section. A crude and adjusted odds ratio with a 95% confidence interval was used to interpret the results. A *P* value of <0.05 indicated statistically significant results.

**Results:**

Of 416 completed charts reviewed, the success rate of vaginal birth after cesarean section was 170 (41%), with 95% CI (36.2%, 45.6%). The factors associated with successful vaginal birth after cesarean section were: macrosomia as past indication of cesarean section delivery: AOR; 0.31, 95% CI (0.15, 0.62); prior successful vaginal birth after cesarean section: AOR; 2, 95% CI (1.18, 3.70); previous successful spontaneous vaginal delivery: AOR; 4, 95% CI (2.05, 7.83); cervical dilatation at admission: AOR; 2.7, 95% CI (1.47, 4.95), and duration of labor: AOR; 1.7, 95% CI (1.07, 2.83).

**Conclusion:**

The success rate of vaginal birth after caesarean section in the study area was low. Macrosomia as past indication of caesarean section, prior vaginal birth after caesarean section, history of vaginal birth, cervical dilatation at admission, and duration of labour were significantly associated with the success rate. Emphasis should be placed on those factors that lead to a higher likelihood of successful vaginal birth.

## 1 Introduction

Caesarean section rates have increased over the last decade, with an estimated third of women having delivered by caesarean section worldwide [1]. In particular, repeat caesarean section is the commonest factor responsible for the overall increased caesarean delivery rates [2]. In Ethiopia, the caesarean delivery rate is increasing [3, 4], which is much higher than the World Health Organizations (WHO) target of a maximum caesarean section rate of 15% [5].

In response to this, efforts have been made to reduce the rates of repeated caesarean delivery, and trial of labour after caesarean section delivery (TOLAC) has been the recommended method as successful vaginal birth after caesarean section (VBAC) is proved to have better maternal and fetal outcome compared with elective repeated caesarean delivery [6] although failed VBAC has an even worst maternal outcome [7]. Careful selection of women who opt for TOLAC remains a wise clinical decision [8].

The proposed rates of successful VBAC have been reported to be 60–80% [9, 10, 11]. However, the VBAC success rate is lower in Africa including Ethiopia [12, 13, 14]. For example successful VBAC was 48% in Nigeria [12], 48% in Ghana [14], and 45.5% in Ethiopia [13] Although successful VBAC is a safer and a more recommended method for women with previous single caesarean section delivery than undergoing elective repeat caesarean delivery [15], the chance of successful VBAC is determined by factors such as history of previous vaginal delivery, cervical dilatation and station at admission, birth weight >4 kg, cephalo-pelvic disproportion, and maternal age [8, 15]; Studies revealed that previous history of vaginal delivery is the best predictor of VBAC success [11, 13, 14].

In Ethiopia, the VBAC rate was 45.5% in Attat Lord Merry Primary Hospital, South Ethiopia [13]. Cervical dilatation >3 cm at admission, history of successful VBAC in the past, rupture of membrane at admission, the occipito-anterior position of the fetus were factors associated with the success rate of VBAC [16].

However, although the rate of caesarean section delivery in Ethiopia is increasing [3, 4], studies on the success rate of VBAC and associated factors in Ethiopia in general and the study area in particular is limited. Therefore, research on the success rate of VBAC and the associated factors among women who gave birth previously by caesarean delivery is needed. Thus, the purpose of this study was to assess the success rate of VBAC and its associated factors in Mizan-Tepi University Teaching Hospital, Southwest Ethiopia.

## 2 Methods and materials

### 2.1 Study design and setting

A facility-based retrospective cross-sectional study was conducted among mothers who gave birth from 2017 to 2019. The study was conducted in Mizan-Tepi University's Teaching Hospital, Mizan-Aman, southwest of Ethiopia. Mizan-Aman is located 569 km away from Addis Ababa, the capital of Ethiopia. Trial of labour, caesarean section, vaginal birth after caesarean section, and evaluations are being performed by general practioners, gynaecologists, and emergency surgeons. The study was conducted from January 6 to February 6, 2020.

### 2.2 Study subjects

The study populations were randomly selected medical charts of registered women who had one previous caesarean section delivery and underwent trial of labour at Mizan-Tepi University Teaching Hospital between 2017 and 2019. Women with one prior lower-segment transverse uterine incision in caesarean section, no previous history of myomectomy, a singleton pregnancy, and no other contraindications to trial of labour were included in this study.

### 2.3 Sample size determination and sampling procedure

The single population proportion formula was used to estimate the required sample size for the study, with the confidence interval estimated to be at the 95% level, the marginal error being 5%, and the proportion of success of VBAC being 45.5%, as observed from a study conducted in Attat Lord Merry Primary Hospital, Southwest Ethiopia [13]; with 10% non-response rate, the final sample size for the study was 419.

A systematic random sampling technique was applied to select patient charts. All charts with one previous caesarean section delivery from 2017 to 2019 were listed based on the sequence of their card number. The sampling interval was calculated by dividing the total patient charts by the estimated sample size (n = 419) leading to a skip interval (k = 3). Then, every 3^rd^ cards were included for data collection.

### 2.4 Operational definitions and variables

**Successful VBAC**: Vaginal delivery of the foetus after undergoing a trail of labour regardless of foetal and maternal complications [17].

**Failed VBAC:** Women who failed to deliver vaginally and ended up with repeat caesarean delivery after undergoing a trail of labour [17].

The dependent variable for the study was vaginal birth after caesarean section (VBAC). Independent variables were maternal age, parity, indication for the previous caesarean section, prior successful VBAC, spontaneous vaginal delivery (SVD), Antenatal care follow-up, history of comorbid medical conditions, antepartum haemorrhage, membrane status at admission, cervical dilatation at admission, duration of labour, and birth weight.

### 2.5 Data collection, method of data analysis, and management

Secondary data, which includes maternal socio-demographic factor (age), reproductive history, past obstetric experience, current obstetric history, and maternal and neonate outcome, were collected using a structured questionnaire from medical charts after tracing the patients’ card numbers. Data were coded and entered into epidata version 3.1.0 and analysed by Statistical Package for Social Science (SPSS) version 21.0 software. Frequency tables, graphs, percentages, mean, and standard deviations were used to summarise the results. Bivariate and multivariable logistic regression analyses were performed to identify the factors associated with the success rate of VBAC. The model goodness-of-fit test was checked using the Hosmer–Lemeshow test (*P* = 0.24), and the model was a good fit. Multicollinearity between the independent variables was checked using the variance inflation factor (VIF<2), and the correlations were acceptable. Crude odds ratio (COR) and adjusted odds ratio (AOR) with corresponding 95% confidence interval were used to interpret the regression results. Statistically significant results were obtained with p < 0.05.

### 2.6 Ethical considerations

This study was approved by the Addis Ababa Medical and Business College Department of general public health ethical review committee with the reference number ERC/00012/2020. A permission letter was also obtained from the study setting. Informed consent was obtained from participants prior to data collection. The study was conducted according to the Declaration of Helsinki involving human subjects.

## 3 Results

### 3.1 Socio-demographic and obstetric characteristics of participants

A total of 419 patient charts were reviewed, resulting in a response rate of 100%. The data analyses were based on 416 completed information collected from patient charts. Two hundred fifty-three (60.8%) of participants were parity three and above. Fifty (12%) of the participants had macrosomia as their past indication of cesarean section. Ninety-four (22.6%) of the participants had a prior history of successful VBAC, and one hundred thirteen (27.2%) participants had cervical dilatation of more than 3 cm at admission in the current pregnancy (Table 1).

**Table 1:** Reproductive and obstetric characteristics of the participants according to their VBAC status at MTUTH, 2020 (n = 416).

Variable	Category	Frequency (%)	VBAC status (n = 416)	
			Success (170, 41%)	Failure (246, 59%)
Parity	I	14 (3.4)	3 (21.4)	11 (78.9)
	II	149 (35.8)	46 (30.9)	103 (69.1)
	≥III	253 (60.8)	121 (47.8)	132 (52.3)
APH	Yes	57 (13.5)	28 (49)	29 (51)
	No	359 (86.5)	142 (39.5)	217 (60.5)
Past indication for CS by macrosomia	Yes	50 (12)	31 (62)	19 (38)
	No	366 (88)	139 (38)	227 (62)
Prior successful VBAC	Yes	94 (22.6)	60 (63.8)	34 (36.2)
	No	322 (77.4)	110 (34.2)	212 (65.8)
Successful SVD at any time point	Yes	196 (47.1)	121 (55)	99 (45)
	No	220 (52.9)	49 (25)	147 (75)
Antenatal care follow-up	Yes	391 (94)	160 (40.9)	231 (59.1)
	No	25 (6)	10 (40)	15 (60)
Membrane status	Ruptured	246 (59.1)	108 (43.9)	138 (56.1)
	Intact	170 (40.9)	62 (36.5)	108 (73.5)
Cervical dilatation	Closed	120 (28.8)	63 (52.5)	57 (47.5)
	<3cm	183 (44.0)	67 (36.6)	116 (63.4)
	≥3cm	113 (27.2)	40 (35.4)	73 (64.6)
Duration of labor	<20 h	283 (68.1)	105 (37.2)	178 (62.8)
	≥20 h	133 (30.9)	67 (50.3)	66 (49.6)
Fetal outcome	Alive	393 (94.5)	156 (39.7)	237 (60.3)
	Dead	23 (5.5)	14 (60.1)	9 (39.9)

Foot note; VBAC; Vaginal Delivery After Caesarean Section; CS: caesarean section; SVD: spontaneous vaginal delivery.

### 3.2 Prevalence of successful VBAC among study participants

Of the 416 women who were on trial of labor from 2017 to 2019 at MTUTH, the prevalence of successful VBAC among the total participants was 41% (N = 170), 95% CI (36.2%, 45.6%) (Table 1).

### 3.3 Factors associated with successful VBAC

#### 3.3.1 Bivariate and multivariate analyses for factors associated with successful VBAC

In multivariable logistic regression analysis, variables such as prior successful VBAC, history of successful SVD at any time point, cervical dilatation, and duration of labour were significantly associated with successful VBAC, whereas fetal macrosomiaas past indication of cesarean section was found to be associated with failure of VBAC (Table 2).

**Table 2:** Bivariate and multivariate logistic regression analyses for factors associated with VBAC at MTUTH, 2020 (n = 416).

Variable	Category	VBAC status (n = 416)		COR (95% CI)	AOR (95% CI)
		Success (%)	Failure (%)		
Parity	I	3 (21.4)	11 (78.9)	3.3 (0.91,12.3)	1 (0.25,4.50)
	II	46 (30.9)	103 (69.1)	2 (1.34,3.14)	1.6 (0.35,7.17
	≥III	121 (47.8)	132 (52.3)	1	1
APH	Yes	28 (50)	28 (50)	1	1
	No	142 (39.5)	217 (60.1)	1.5 (0.86,2.68)	1.3 (0.67,2.55)
Macrosomia	Yes	31 (62)	19 (38)	0.37 (0.20,0.69)	0.31 (0.15,0.62)
	No	139 (38)	227 (62)	1	1
Prior successful VBAC	Yes	60 (63.8)	34 (36.2)	3.4 (2.10,5.49)	2 (1.18,3.70)
	No	110 (34.2)	212 (65.8)	1	1
Successful SVD at any time point	Yes	121 (55)	99 (45)	3.6 (2.41,5.57)	4 (2.05, 7.83)
	No	49 (25)	147 (75)	1	1
ANC follow-up	Yes	160 (40.9)	231 (59.1)	0.9 (0.42,2.19)	0.9 (0.42,2.19)
	No	10 (40)	15 (60)	1	1
Membrane status	Ruptured	108 (43.9)	138 (56.1)	0.7 (0.49,1.09)	0.6 (0.40,1.03)
	Intact	62 (36.5)	108 (73.5)	1	1
Cervical dilatation	Closed	63 (52.5)	57 (47.5)	1	1
	<3cm	67 (36.6)	116 (63.4)	1.9 (1.19,3.05)	2.3 (1.36, 4.09)
	≥3cm	40 (35.4)	73 (64.6)	2 (1.19,3.41)	2.7 (1.47, 4.95)
Duration of labour	<20 h	104 (37.4)	174 (62.6)	1.7 (1.16,2.72)	1.7 (1.07,2.83)
	≥20 h	66 (51.5)	62 (48.5)	1	1
Fetal outcome	Alive	156 (39.7)	237 (60.3)	2.3 (0.99,5.59)	1.3 (0.50,3.83)
	Dead	14 (60.1)	9 (39.9)	1	1

Foot note; VBAC; Vaginal Delivery after Caesarean Section; APH: Ante partum hemorrhage; SVD: spontaneous vaginal delivery; ANC: Ante natal care.

Women whose previous indication for cesarean section delivery due to fetal macrosomia as past indication of cesarean delivery were 69 % less likely to undergo successful VBAC than women whose previous indication for CS delivery was not due to fetal macrosomia: AOR; 0.31, 95% CI (0.15, 0.62). Women who had prior successful VBAC were two times more likely to have successful VBAC than those who had no prior successful VBAC: AOR; 2, 95% CI (1.18, 3.70). Those women with a history of successful spontaneous vaginal delivery before were four times more likely to undergo successful VBAC than women with no history of successful spontaneous vaginal delivery: AOR; 4, 95% CI (2.05, 7.83).

Women with cervical dilatation of less than 3 cm at admission were 2.3 times more likely to undergo successful VBAC than women with a closed cervix at admission: AOR; 2.3, 95% CI (1.36, 4.09). Similarly, women with cervical dilatation ≥3 cm at admission were 2.7 times more likely to undergo successful VBAC than women with a closed cervix at admission: AOR; 2.7, 95% CI (1.47, 4.95). Women whose labour took less than 20 h were 1.7 times more likely to undergo successful VBAC than women whose labour duration was more than 20 h: AOR; 1.7, 95% CI (1.07, 2.83).

## 4 Discussion

This study was conducted to assess the success rate of VBAC and the associated factors in Mizan-Tepi University's Teaching Hospital. Completed data were reviewed from charts among women who gave birth in Mizan-Tepi University's Teaching Hospital between 2017 and 2019. This study found that the prevalence of successful VBAC among study participants was 170 (41%), 95% CI (36.2%, 45.6%). Fetal macrosomia as past indication of cesarean section delivery, prior successful VBAC, history of spontaneous vaginal delivery at any time point, cervical dilatation at admission, and duration of labour were significantly associated with the success rate of VBAC.

Out of the total 416 completed charts, the prevalence of successful VBAC among study participants was 170 (41%), 95% CI (36.2%, 45.6%). This finding is consistent with similar studies conducted before in Ethiopia and other countries, which reported that the success rate of VBAC was 44.5% at Attat Lord Merry Primary Hospital, Gurage Zone, and South Ethiopia in 2018 [13], and 41.5% in Bahrain in 2017 [18].

However, the current VBAC success rate is lower than that reported in a study conducted in Addis Ababa in 2013, which revealed a VBAC success rate of 49% [19]. A success rate of 63.3 % was reported in a study conducted at Felegehiwot Referral Hospital, Northwest Ethiopia, in 2015 [20], 50% was reported in Nigeria in 2014 [21], 51.5% was reported in Thailand in 2018 [17], 72% was reported in a study conducted in Egypt in 2014 [22], and 84.9% was reported in Taiwan in 2017 [23]. On the other hand, the rate reported in the current study is higher than that reported in the study conducted in Pakistan which reported that the prevalence of VBAC success was only 34% [24]. The difference might be due to differences in hospital settings or protocols for trial of labor after caesarean section across countries, which of course might vary depending on the facility that the hospitals possessed. For instance, the setting from which the current study being conducted is somehow less equipped than other settings where advanced labor and delivery facilities are available. This might influence physicians to opt for elective cesarean section delivery for women who had delivered with caesarean section previously for fear of nonreassuring fetal heart rate and other complications that would put the mother and child's health more at risk. This might explain the observed inconsistencies between those similar studies. The difference in population characteristics and the time gap between studies might also explain the observed gaps between the current study and other studies being compared here.

With regard to factors associated with the success rate of VBAC, this study found that women who had previous indication of Caesarean delivery by fetal macrosomia were less likely to undergo successful VBAC: AOR; 0.31, 95% CI (0.15, 0.62).This is consistent with a systematic review that reported that birth weight >4000 g reduces the likelihood of successful VBAC (AOR; 0.56; 95% CI: 0.50–0.64) [25]. Therefore, it is appropriate that candidates of trial of labor after caesarean section should be evaluated for their previous birth weight to increase the likelihood of VBAC success.

Prior history of VBAC was also significantly associated with the success rate of VBAC in the current study: AOR; 2, 95% CI (1.18, 3.70). Similar findings were reported by previous studies conducted in different time periods and places [13, 17]. For example, a study conducted in Attat Lord Merry Primary Hospital, Gurage Zone, and South Ethiopia revealed that women with a prior history of VBAC were more likely to undergo successful VBAC than those without prior history of vaginal birth after caesarean section [13]. In China, successful VBAC was highest (96.4%) among women with history of vaginal delivery (11). Therefore, obstetricians should encourage women with previous history of vaginal delivery to TOLAC. In women with a history of successful spontaneous vaginal delivery at any time point had a higher likelihood of successful VBAC than their counterparts: AOR; 4, 95% CI (2.05, 7.83).This finding is in line with that of another similar study conducted in Thailand in 2017 which reported that the history of prior vaginal birth was significantly associated with the success rate of VBAC: AOR; 3.17, 95% CI (1.87–5.36) [17].

Cervical dilatation at admission was associated with increased likelihood of successful VBAC compared to no cervical dilatation. Similar findings were reported that support that women with cervical dilatation at admission were more likely to experience successful VBAC than women without cervical dilatation in Ethiopia [13]. In Egypt, a similar finding was reported [22]. The duration of labour was also found to have a significant association with the success rate of VBAC in the current study. Women whose labor took less than 20 h were more likely to experience successful VBAC than women whose labor duration was more than 20 h: AOR; 1.7, 95% CI (1.07, 2.83). This is consistent with the findings of other studies that report that the duration of the active stage of labor is significantly associated with the success rate of VBAC [22].

**Strength and limitation:** The representative sampling method was used and it's possible to generalize the finding to a similar setting in Ethiopia. Since a secondary data source (patient chart) was used, some important demographic and clinical variables might not be included in the analysis. This might affect the association of those missing variables with the dependent variable (success rate of VBAC). The cross-sectional nature of the study design used in this study also makes it difficult to know the temporal associations.

## 5 Conclusion

The prevalence of the success rate of VBAC among study participants in the study area was low. Fetal macrosomia, prior history of VBAC, history of spontaneous vaginal delivery, cervical dilatation at admission, and duration of labour were significantly associated with successful VBAC. Vaginal birth after caesarean section would be a viable option to decrease caesarean section rates in Ethiopia, and gynaecologists or other health care providers should consider the identification of factors among women that lead to a higher likelihood of successful vaginal birth after caesarean section. Future studies should consider prospective study design using a primary data source.

## Declarations

### Author contribution statement

Yeabsira Girma and Zerihun Menlkalew: Conceived and designed the experiments; Performed the experiments; Wrote the paper.

Alemnew Destaw: Analyzed and interpreted the data; Contributed reagents, materials, analysis tools or data; Wrote the paper.

### Funding statement

This research did not receive any specific grant from funding agencies in the public, commercial, or not-for-profit sectors.

### Data availability statement

Data will be made available on request.

### Declaration of interests statement

The authors declare no conflict of interest.

### Additional information

No additional information is available for this paper.
